# Multistage machine learning model for automated referral triage in pain medicine

**DOI:** 10.1016/j.fhj.2026.100500

**Published:** 2026-01-06

**Authors:** Lan Jiang, Yu-Li Huang, Matthew J. Pingree, Mark A. Bendel

**Affiliations:** a Center for the Science of Health Care Delivery, Mayo Clinic, Rochester 55905, MN, United States; b Department of Anesthesiology, Mayo Clinic, Rochester 55905, MN, United States

**Keywords:** Referral triage, Multistage machine learning, Pain medicine, Elbow method

## Abstract

Effective referral triage in pain medicine is essential to ensure that patients receive timely and appropriate care. This study presents a multistage machine learning framework to better identify patients who may benefit from one of five specialised pain procedures. Two years of clinic data were used for training and validation, consisting of 231 features for 3,552 patients. The proposed approach started with the baseline model selection, followed by stage iterations acquiring the elbow method for stage decision. The Easy Ensemble was selected among methods and applied for each stage. Results showed that the approach improves prediction accuracy in the true positive rate (TPR) and area under the curve (AUC). The final-stage models achieved the improvement over single-stage model by as much as 34.8% and 23% on TPR and AUC. This multistage framework can enhance triage accuracy and hold potential for broader application in other clinical settings.

## Highlights

• The multistage machine learning method shows improved performance over traditional single-stage models, delivering higher accuracy in referral triage in pain medicine.
• By enabling more accurate triage in pain medicine, the proposed method ultimately reduces unnecessary visits, enhances overall healthcare efficiency and improves patient care.
• The proposed framework is adaptable beyond pain medicine and holds strong potential for improving clinical workflows across other medical departments.

## Introduction

Advanced pain management procedures, such as basivertebral radiofrequency ablation (BVRF), spinal cord stimulation (SCS), peripheral nerve stimulation (PNS), intrathecal pump (ITP) and minimally invasive lumbar decompression (MILD), are increasingly performed in the department of pain medicine (PM). They provide much tailored pain management for patient-specific conditions.^1^ Patients are usually referred to PM for various pain-related issues from other specialties like spine centre and oncology, and scheduled with the first available provider with limited or no triaging on patients’ needs. It often leads to redundant appointments, delays in care, and inefficiencies within healthcare operations. Despite efforts made to predetermine the most appropriate pain management services for patients, triaging patients at the time of referral requests remains challenging due to the absence of a universal gold standard. Most PM practices rely on the judgement and experience of providers to make treatment decisions, which results in care inconsistency.

Existing literature on referral triaging for patient treatments in PM remains limited. A few triage tools have been developed with notable limitations. One tool was designed for screening candidates for SCS based on inclusion and exclusion criteria, targeting patients with chronic lower back and leg pain.^2^ Its narrow focus limits generalisability across other pain conditions and treatment options. A trial of multidisciplinary team conference process involving both PM specialists and psychologists has been explored to assist in selecting candidates for an SCS procedure or a dorsal root ganglion (DRG) stimulation.^3^ This approach offers the advantage of incorporating diverse clinical perspectives, but it is resource-intensive and difficult to scale. A study developed an automated triage system utilising machine learning methods to identify patients who may benefit from an SCS procedure.^4^ While this tool provides a valuable pipeline for integration with electronic health record (EHR) systems, its scope remains confined to SCS candidates. These efforts highlight important steps towards improving triage processes, but also underscore the ongoing need for more comprehensive, scalable and generalisable solutions.

With advancements in technology, machine learning has become an increasingly valuable method for prediction in healthcare. Research on machine learning in referral triaging highlights progress in streamlining triage and mostly focus on prioritising patients’ needs,^5^, ^6^, ^7^ rather than guiding them to the appropriate care path. While making substantial progress in such technology, ongoing research continuously explores methods to enhance model performance, addressing limitations in accuracy, robustness and generalisability across diverse applications. Ensemble methods have emerged as effective strategies to improve performance by integrating the strengths of multiple models. For instance, stacked generalisation combines predictions from diverse base learners^8^ to improving accuracy in severe cardiac clinical outcome.^9^ Similarly, boosting methods, such as AdaBoost^10^^,^^11^ and Gradient Boosting,^12^^,^^13^ iteratively refine models focusing on misclassified instances. It has been explored in pain medicine triage,^4^ disease classification^14^^,^^15^ and medical treatment prediction.^16^ In addition, cascade forest, a non-neural deep learning method, mimics deep learning depth but with simpler models^17^ and shows promising results in disease classification.^18^, ^19^, ^20^ However, there is limited literature on iteratively adding prediction results as new features in a multistage machine learning framework for model refinement.

This study proposes a multistage machine learning approach that triages patients to a diverse range of procedures offered in PM by incorporating results from earlier stages into subsequent stages to iteratively refine its decision-making process, improving prediction performance.

## Methods

### Data description

The study focuses exclusively on patients referred internally from other departments to the PM department in the institution. Two years of referrals, from June 2021 to June 2023, were collected for a total of 3,552 patients. Among these patients, 46 patients underwent PNS, 126 patients received SCS, 36 patients had ITP, 16 patients received BVRF, and 10 patients underwent MILD procedures. These procedures were treated as outcomes for our predictive models, which exhibited highly imbalanced characteristics. The input features included both structured data (192 features) and unstructured data such as clinical notes (39 features) for a total of 231 features; shown in Appendix Table 1. Structured features captured patients’ medical history within 1 year prior to the referral date, whereas unstructured features reflected information documented within 90 days. The structured data represented patient characteristics, referral diagnosis, medications, relevant appointments histories and comorbidities. The unstructured data were based on rule-based natural language processing (NLP) contexts^21^ used to extract concepts mentioned in the clinical notes, including symptoms and past procedures, especially from the referral. The NLP model used two types of rules: text rules and context rules. Text rules identified keywords and phrases related to each target concept, while context rules helped detect negations and distinguished whether a condition concerning the patient or someone else, such as family history. Both rules are validated by PM providers to ensure that the extracted information was accurate and useful.

**Table 1:** Results of baseline machine learning models.

Models	PNS			SCS			ITP			BVRF			MILD			Overall range		
	AUC	TPR	TNR	AUC	TPR	TNR	AUC	TPR	TNR	AUC	TPR	TNR	AUC	TPR	TNR	AUC	TPR	TNR
Easy Ensemble	0.75	64%	76%	0.81	68%	80%	0.94	89%	88%	0.84	83%	66%	0.95	80%	84%	(0.75,0.95)	(64%,89%)	(66%,88%)
Balanced Bagging	0.75	47%	83%	0.79	53%	84%	0.95	84%	89%	0.84	67%	84%	0.95	80%	91%	(0.75,0.95)	(47%,84%)	(83%,91%)
RUS Boost	0.71	45%	78%	0.80	58%	82%	0.96	76%	94%	0.81	67%	68%	0.96	90%	82%	(0.71,0.96)	(45%,90%)	(68%,94%)
Random forest	0.72	0%	100%	0.78	46%	85%	0.94	3%	100%	0.74	0%	100%	0.88	30%	99%	(0.72,0.94)	(0%,46%)	(85%,100%)
Balanced Random Forest	0.72	66%	74%	0.80	71%	74%	0.95	87%	82%	0.68	61%	75%	0.89	80%	82%	(0.68,0.95)	(61%,87%)	(74%,82%)
Logistic regression	0.67	40%	90%	0.76	51%	85%	0.87	63%	96%	0.79	61%	86%	0.89	70%	93%	(0.67,0.89)	(40%,70%)	(85%,96%)
L1 logistic regression	0.64	40%	91%	0.74	53%	85%	0.84	61%	96%	0.82	56%	89%	0.86	60%	95%	(0.64,0.86)	(40%,61%)	(85%,96%)
L2 logistic regression	0.67	40%	90%	0.76	51%	85%	0.87	63%	96%	0.79	61%	86%	0.89	70%	93%	(0.67,0.89)	(40%,70%)	(85%,96%)
One-Class SVM	0.51	85%	15%	0.64	91%	12%	0.53	92%	3%	0.54	6%	91%	0.88	80%	80%	(0.51,0.88)	(6%,92%)	(3%,91%)
SVC	0.71	28%	94%	0.78	50%	85%	0.89	55%	96%	0.80	72%	80%	0.91	90%	82%	(0.71,0.91)	(28%,90%)	(80%,96%)

### Baseline machine learning models

Several baseline machine learning methods were selected to address class imbalance and provide initial comparisons. These methods include ensemble techniques, such as Easy Ensemble,^22^ Balanced Bagging,^23^ RUS Boost^24^ and Balanced Random Forest,^25^ which combine resampling strategies to improve minority class prediction. Additionally, weighted versions of traditional models, such as random forest^26^ and logistic regression,^30^ were employed to adjust the influence of each class during training. Both L1 and L2 regularised logistic regression models with balanced class weighted distributions were included to assess the impact of regularisation under imbalanced conditions. Finally, One-Class Support Vector Machine (One-Class SVM)^27^ and standard Support Vector Classifier (SVC)^28^ were explored as alternative approaches to capture the underlying structure of rare events. Appendix Table 2 provides a summary of the strengths and weaknesses of the models. All models were coded in Python using packages, imbalanced-learn^29^ and scikit-learn.^30^ The performance metrics include area under the curve (AUC), true positive rate (TPR) and true negative rate (TNR). The goal was to select a baseline model that consistently performed well on all three metrics for the next step.

**Table 2:** Summary of final stage results.

	ITP	SCS	MILD	PNS	BVRF
Precision	0.16	0.13	0.13	0.08	0.09
Recall	0.97	0.87	1.00	0.96	1.00
F1-score	0.27	0.23	0.23	0.14	0.17
AUC (mean)	0.9861 ± 0.0089	0.8944 ± 0.0290	0.9913 ± 0.0025	0.9593 ± 0.0282	0.9926 ± 0.0071
95% CI for AUC	[0.9798, 0.9925]	[0.8737, 0.9151]	[0.9895, 0.9930]	[0.9391, 0.9794]	[0.9876, 0.9977]

### Multistage machine learning approach

The multistage method is an iterative machine learning framework designed to enhance model performance by incorporating prediction outcomes from earlier stages into subsequent processes with an iteration-stopping criterion. Thus, at each stage, the model learns from both the features from the original data and the additional features generated by the prediction results from the previous stages, allowing it to refine its understanding over time. This iterative process helps the model recognise more complex patterns and improve performance with each stage. A 10-fold cross-validation is then applied at each stage to prevent overfitting or data leakage, ensuring that improvement validity.^31^

**Fig. 1:**
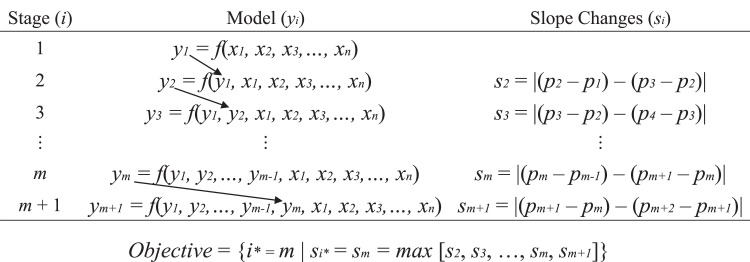
Multistage machine learning framework.

The iterative machine learning framework is illustrated in Fig. 1. Let *y_i_* be the prediction outcomes in terms of prediction probability at stage *i*, where *i* = 1,2, …,*m,m*+1. Let *x_j_* be the features collected from the databases, both structured and unstructured data, where *j* = 1,2,…,*n. y_1_* is the initial model outcome or stage 1 model probability. *y_2_* is the stage 2 model probability that incorporates *y_1_* as an additional feature along with all *x_j_*. Let *s_i_* be absolute value of slope changes between stage *i*-1 to *i* and *i* to *i*+1. The slope represents the magnitude of the model performance improvement made at each stage *i*, particularly the TPR. Let *p_i_* be the TPR at stage *i*. Hence, *s_i_* = |(*p_i_*–*p_i_*_-1_)–(*p_i_*_+1_–*p_i_*)|. The iteration continues until *s_i_ = s_m_ = max*[*s_2_,s_3_*,…,*s_m_,s_m+1_*] where *s_i_* is optimal at *i* = *m* = i*. The concept of slope changes is also known as the elbow method, which is used to determine the optimal number of clusters for a dataset,^32^^,^^33^ or that of eigenvalues for principal components.^34^^,^^35^ This approach aims to iteratively leverage accumulated information, resulting in improved predictive performance.

## Results

### The baseline machine learning model selection

The results of baseline machine learning models are summarised in Table 1. Table 1 reports AUC, TPR and TNR for each procedure as well as the range of these among procedures. For this study, we primarily focused on TPR while AUC and TNR were reasonable. The results indicated that Easy Ensemble performed consistently better than others for all procedures in TPR and AUC. It achieved TPRs ranging from 64% to 89%, with AUCs between 0.75 and 0.95, and TNRs from 66% to 88%. Other methods such as Balanced Random Forest also performed well, with comparable AUCs (0.68–0.95), and balanced TPR (61%–87%) and TNR (74%–82%) across procedures. Traditional logistic regression models and support vector classifiers demonstrated higher TNRs but substantially lower TPRs, indicating a bias towards negative predictions. Notably, One-Class SVM showed extremely high TPRs but very poor TNRs, making it unsuitable for clinical decision-making. The results also reveal that no single method consistently performed well across all procedures. This highlights the complexity of the classification task and suggests the need for more advanced or tailored approaches. Based on the results, Easy Ensemble was selected for the proposed multistage method.

### The results of multistage machine learning approach

**Fig. 2:**
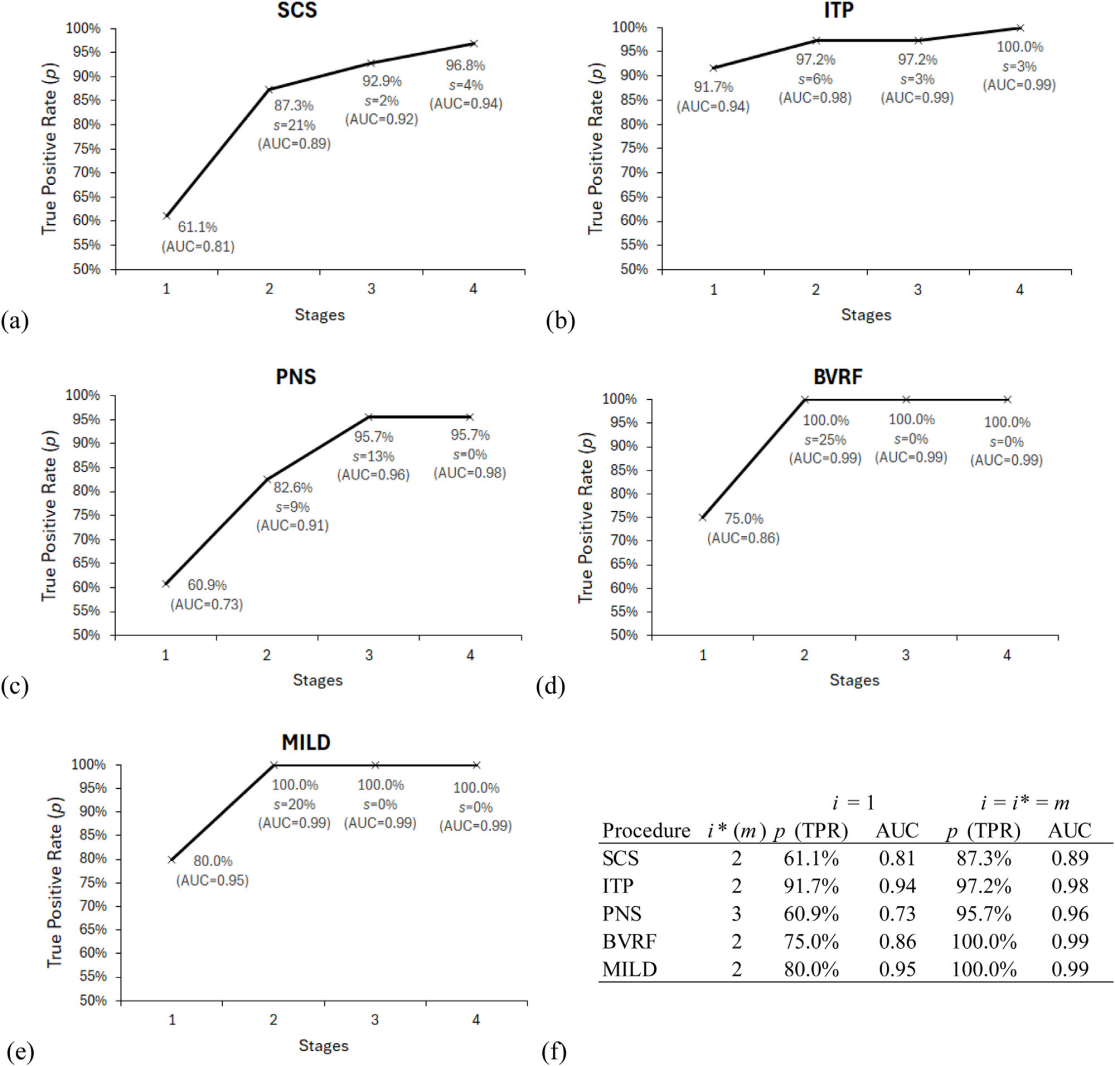
The multistage machine learning decision using elbow method for procedure (a) SCS, (b) ITP, (c) PNS, (d) BVRF, (e) MILD, and (f) summary of each procedure.

Easy Ensemble was then applied to the multistage machine learning approach with the goal of improving TPR. The resulting number of machine learning stages for each procedure were reported as well as prediction performance; see Fig. 2. Four stages for each procedure were demonstrated along with their TPRs, slope change percentage and AUC. Fig. 2(a) indicated that for SCS, the slope changes the most at 21% in the second stage, resulting a TPR of 87.3% and an AUC of 0.89. Fig. 2(b) indicated that for ITP, the slope changes the most at 6% in the second stage, resulting in 97.2% TPR and 0.98 AUC. Fig. 2(c) indicated that for PNS, the slope changes the most at 13% in the third stage, resulting in 95.7% TPR and 0.96 AUC. Fig. 2(d) indicated that for BVRF, the slope changes the most at 25% in the second stage, resulting in a 100% TPR and 0.99 AUC. Fig. 2(e) indicated that for MILD, the slope changes the most at 20% in the second stage, resulting in 100% TPR and an 0.99 AUC. The summary in comparison between one-stage and multistage machine learning is shown in Fig. 2(f).

**Fig. 3:**
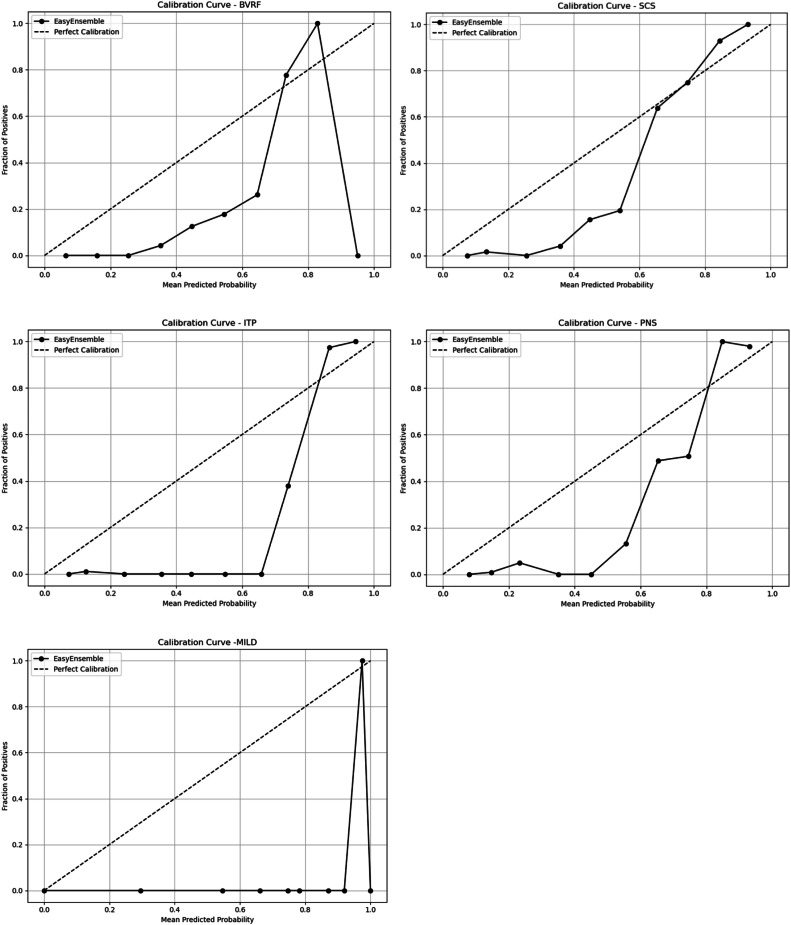
Calibration plots.

Additionally, Table 2 and Fig. 3 report multistage model results, including precision, recall, F1-score, 95% CI for AUC and calibration plot. While precision was modest (from 0.08 to 0.16), recall remained consistently high (0.87–1.00), indicating the model’s ability to correctly identify patients for each procedure. Procedures MILD and BVRF had very few positive cases (*n* = 10 and *n* = 16, respectively), which could limit model stability and inflate AUC estimation. The F1-scores (0.14–0.27) demonstrated the trade-off between high sensitivity and lower precision, which was a common characteristic when dealing with imbalanced data. The calibration plot of each procedure generally fell below the perfect calibration line across most probability bins. This reflects the reality that many bins contain few or no positive cases due to highly imbalanced data. The higher predicted probability bins showed values rising above the perfect line, as these bins contained the observed positive cases aligned with model predictions. This pattern was expected in a setting with very low event rates.

### Stage performance for each procedure

The multistage approach was proven to be effective over a single-stage model. To further demonstrate its impact, a detailed insight was provided; see Table 3. Among 3,552 total cases, there were 126 positive SCS cases. The two-stage model accurately predicted 77 out of 126 cases at stage 1. For the other 49 cases, the second stage was able to project 33 additional cases without making mistakes on the initial 77 cases. For the negative cases, although stage 2 was able to pick up 45 more cases from the mistake at stage 1, it altered 103 cases that were accurately predicted from stage 1. For ITP, the two-stage model missed only three out of 36 positive cases at stage 1, and 2 out of these three were captured by stage 2. As for negative cases, out of 500 cases that were missed by stage 1, 312 cases were accurately identified by stage 2. For PNS, the three-stage model predicted 28 positive cases at stage 1, 10 additions from stage 2, and six more at stage 3. For negative cases, 372 cases missed by stage 1 were captured by stage 2, and 112 out of 581 cases missed by both stage 1 and 2 were captured by stage 3. Also, 128 out of 184 cases that were changed incorrectly by stage 2 from stage 1 were captured by stage 3. Both BVRF and MILD were determined to be the two-stage model. For positive cases, cases missed by stage 1 were all captured by stage 2. For negative cases, 800 out of 957 BVRF cases and 505 out of 568 MILD cases were additionally captured by stage 2.

**Table 3:** Positive and negative cases stage performance for each procedure.

## Discussions

The results summarised the multistage approach performance and highlighted the significant improvements over the single stage model across all five advanced PM procedures. The predicted probability from the previous stage serves as a score of what the model has already learned. Adding this score as a new feature to the next stage can refine its decisions, resulting in better performance. This strategy is conceptually like the iterative refinement techniques used in deep learning architectures, where predictions are progressively improved by leveraging information from earlier stages. These improvements allow the PM department to schedule patients with the most appropriate provider, reducing unnecessary appointments and increasing patient access to care. Based on the findings, the model could potentially identify 24% more positive patients compared to the initial stage. Moreover, the proposed approach can potentially be extended to other areas of pain management, including non-advanced interventional procedures like epidural steroid injections and non-interventional programmes such as pain rehabilitation with future investigations. By incorporating a broader range of procedures and programmes, the proposed framework could further enhance automated triage across the full range of PM services.

### Implementation

**Fig. 4:**
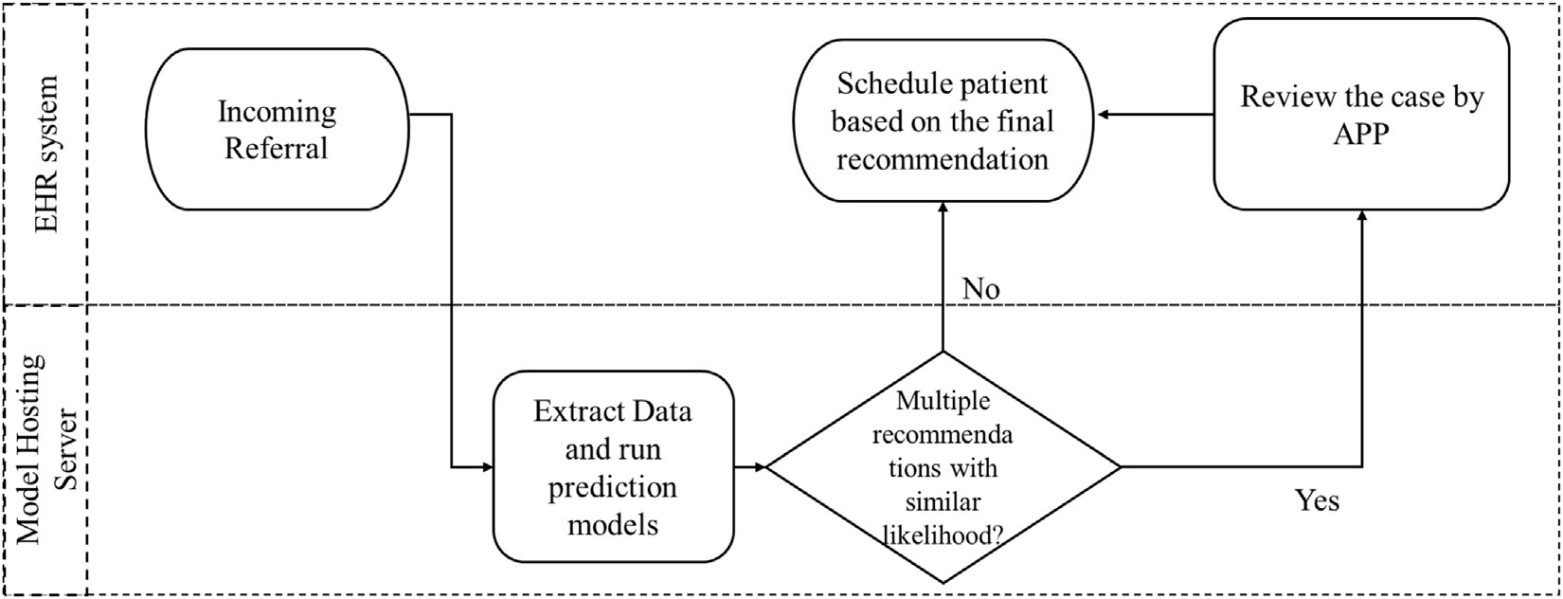
System pipeline overview.

Fig. 4 provides a high-level overview of implementation pipeline. After incoming referrals are created in the EHR system, the relevant data will be automatically extracted and processed by the model hosted in a server. When a patient referral results in multiple potential outcomes, the procedure with the highest predicted probability will be selected for scheduling. However, when the probabilities are very close, the case will be flagged for manual review. In these cases, advanced practice providers (APPs) such as nurse practitioners or physician assistants are notified to review the results and make the final decision before sending them to the scheduling queue. This human-in-the-loop approach helps to balance automation efficiency with clinical oversight. As this model served as a decision support tool at the initial phase, recommendations with top ten features (reported in Supplementary Materials Top 10 Features of Final Stage Models) will be populated and reviewed by APPs, ensuring that every recommendation is safeguarded by human oversight. In addition, the model is planned to be retrained every 6 months to account for changes in patient patterns, clinical practices and referral behaviours. This phased approach allows the system’s real-life performance, calibration and clinical relevance to be monitored and improved before fully integration into automated triage workflows.

### Elbow method decision on optimal number of stages

**Fig. 5:**
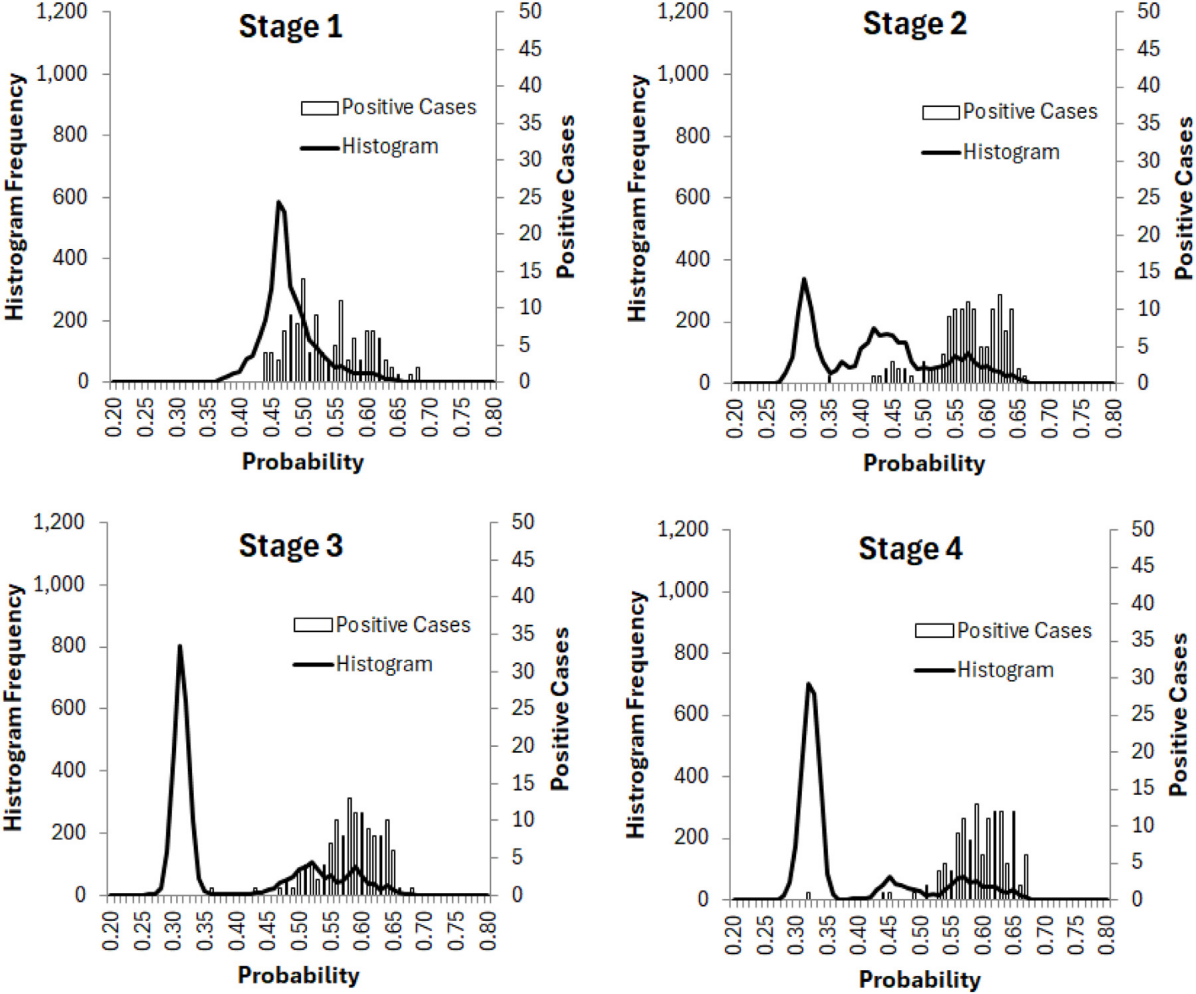
Predictive probability distribution and positive cases allocation for SCS procedure.

The elbow method prevented the decision from overfitting.^36^^,^^37^ In our study, we determined the number of machine learning stages to finalise the training model. Using the SCS procedure as an example, we could continue adding stages and both TPR and AUC would continue to increase; see Fig. 2(a). At stage 4, TPR reached 96.8% and AUC reached 0.94. Fig. 5 presented how SCS predictive probabilities and positive cases were distributed. The probability histogram started with an approximate normal distribution with a mean slightly <0.5 at stage 1. Then the distribution began shifting to the left, but a small portion trended to the right at stage 2. This indicated that a possible separation of data led to multimodal formation. By stage 4, two small modes and one large peak were formed. With the probability cut-off at 0.5, the trend of positive cases started moving to the right, which increased the likelihood of positive cases being greater than 0.5 and resulted in higher TPR. We believed that somewhere between stage 1 and 4, an overfitting occurred. Therefore, the elbow method stopped the overfitting and decided the optimal number of machine learning stages at two for the SCS procedure. The term ‘overfitting’ has been justified loosely in data modelling society. A further investigation is needed to define the term better.

### Limitations

The multistage approach introduces certain limitations. First, the use of multiple sequential models increases the computational complexity and processing time compared to single-model approaches. Second, while this study focuses on Easy Ensemble due to its performance, a broader range of machine learning models should be explored and could yield additional insights for further improvement. Third, the interpretation of the added features from previous stages can be challenging, particularly when applying the model in clinical practice. Fourth, some models trained on extremely small positive sample sizes, such as MILD and BVRF, may show inflated accuracy and such performance may not be reliable. Fifth, this study relies on confirmed procedural records from the electronic medical record system as a reproducible proxy outcome, recognising that such records may not fully represent the appropriateness of the intervention due to variability in clinical decision-making and patient preferences. Lastly, any patient population or provider documentation behaviour changes will affect the model performance; see Supplementary Material Additional Testing. Hence, performance monitoring and model retraining are required, which can be burdensome. Despite these considerations, the multistage framework offers a promising pathway for improving prediction accuracy in complex clinical decision-making tasks.

## Conclusion

This study introduced a multistage machine learning framework designed to support a more accurate triage decision for a patient who may benefit from a targeted pain management procedure. The key concept behind this approach is to build predictions in stages: each stage uses not only the original patient data, but also the prediction results from the previous stage. By adding this extra layer of information, the model becomes more informed and, as a result, more accurate. The novelty of the proposed approach provides a proof-of-concept using the data from the participating PM department. Further validation and real-world testing are required before being used in patient triage as a clinical decision support tool for its adaptability to other areas of medicine.

## Ethics approval and consent to participate

Our institutional review board (IRB) acknowledges that based on the responses submitted for this study through the Human Subjects Research Wizard tool, and in accordance with the Code of Federal Regulations, 45 CFR 46.102, the study does not require IRB review. No identifiable or individualised patient data were used. All methods were carried out in accordance with relevant guidelines and institutional policies.

## Data availability statement

The data that supports the findings of this study are available from the corresponding author upon reasonable request.

## CRediT authorship contribution statement

**Lan Jiang:** Writing – review & editing, Writing – original draft, Visualization, Validation, Software, Methodology, Formal analysis, Data curation, Conceptualization. **Yu-Li Huang:** Writing – review & editing, Writing – original draft, Visualization, Validation, Supervision, Project administration, Methodology, Investigation, Formal analysis, Conceptualization. **Matthew J. Pingree:** Writing – review & editing, Conceptualization. **Mark A. Bendel:** Writing – review & editing, Supervision, Conceptualization.

## Declaration of competing interest

The authors declare that they have no known competing financial interests or personal relationships that could have appeared to influence the work reported in this paper

## Appendix Supplementary materials

Image, application 1

Image, application 2
