# Supplementary material for: Multistage machine learning model for automated referral triage in pain medicine
Source: Future Healthc J. 2026 Jan 6;13(1):100500. doi: 10.1016/j.fhj.2026.100500 (PMC12860341; doi:10.1016/j.fhj.2026.100500)
Supplement: Supplementary file 1 [file mmc1.docx]

Appendix Table 1 Data features

| **General** |  |
| --- | --- |
| **Factors** | **Description** |
| age | age |
| Gender | Gender |
| in hospital | in hospital (yes/no) |
| **Surgery, Appointment and Medication History** |  |
| **Factors** | **Description** |
| procs_n_Cancer | Number of Cancer Procedures |
| procs_n_Other | Number of Other Procedures |
| procs_n_PNS | Number of PNS Procedures |
| procs_n_PRC | Number of PRC Procedures |
| Procs_n_SCS | Number of SCS Procedures |
| surgeries_n_Orthopedic.Surgery | Number of Orthopedic Surgeries |
| surgeries_n_Other | Number of Other Surgeries |
| other_appts_n_Palliative.Medicine | Number of Appointments w/ Palliative Medicine |
| other_appts_n_Radiation.Oncology | Number of Appointments w/ Radiation Oncology |
| other_appts_n_Psychiatry.and.Psychology | Number of Appointments w/ Psychiatry and Psychology |
| other_appts_n_Infusion.Therapy | Number of Appointments w/ Infusion Therapy |
| other_appts_n_Anesthesiology | Number of Appointments w/ Anesthesiology |
| other_appts_n_Radiology | Number of Appointments w/ Radiology |
| other_appts_n_Neurology | Number of Appointments w/ Neurology |
| other_appts_n_Physical.Medicine.and.Rehabilitation | Number of Appointments w/ Physical Medicine and Rehabilitation |
| other_appts_n_Orthopedic.Surgery | Number of Appointments w/ Orthopedic Surgery |
| other_appts_n_Neurological.Surgery | Number of Appointments w/ Neurological Surgery |
| other_appts_n_Spine | Number of Appointments w/ Spine |
| other_appts_n_Oncology | Number of Appointments w/ Oncology |
| other_appts_n_Other.Surgery | Number of Appointments w/ Other Surgery |
| other_appts_n_Other | Number of Appointments w/ Other |
| orders_n_Amitriptyline | Number of Medication Order for Amitriptyline |
| orders_n_Antibiotic | Number of Medication Order for Antibiotic |
| orders_n_Benzodiazepine | Number of Medication Order for Benzodiazepine |
| orders_n_Blood.Thinner | Number of Medication Order for Blood.Thinner |
| orders_n_Cyclobenzaprine | Number of Medication Order for Cyclobenzaprine |
| orders_n_Duloxetine | Number of Medication Order for Duloxetine |
| orders_n_Gabapentin | Number of Medication Order for Gabapentin |
| orders_n_Nortriptyline | Number of Medication Order for Nortriptyline |
| orders_n_Opioid | Number of Medication Order for Opioid |
| orders_n_Other | Number of Medication Order for Other |
| adt_n | Number of Hospitalizations |
| **Hierarchical Condition Category History (HCC)** |  |
| **Factors** | **Description** |
| dx_n_1 | Acute Renal Failure |
| dx_n_2 | all other |
| dx_n_3 | Bone Joint Muscle Infections Necrosis |
| dx_n_4 | Breast Prostate and Other Cancers and Tumors |
| dx_n_5 | Cardiorespiratory Failure and Shock |
| dx_n_6 | Chronic Kidney Disease Mild / Unspecified / Stages 1 or 2 / Unspecified |
| dx_n_7 | Colorectal Bladder and Other Cancers |
| dx_n_8 | Diabetes with Acute Complications |
| dx_n_9 | Diabetes with Chronic Complications |
| dx_n_10 | Diabetes without Complication |
| dx_n_11 | Disorders of Immunity |
| dx_n_12 | Hip Fracture Dislocation |
| dx_n_13 | Intestinal Obstruction Perforation |
| dx_n_14 | Lung and Other Severe Cancers |
| dx_n_15 | Lymphoma and Other Cancers |
| dx_n_16 | Major Depressive Bipolar and Paranoid Disorders |
| dx_n_17 | Metastatic Cancer and Acute Leukemia |
| dx_n_18 | Other pain |
| dx_n_19 | Palliative Care Hepatocellular Carcinoma |
| dx_n_20 | Polyneuropathy |
| dx_n_21 | Rheumatoid Arthritis and Inflammatory Connective Tissue Disease |
| dx_n_22 | Spinal Cord Disorders Injuries |
| dx_n_23 | Vascular Disease |
| dx_n_24 | Vascular Disease with Complications |
| dx_n_25 | Vertebral Fractures without Spinal Cord Injury |
| **Diagnosis history** |  |
| **Factors** | **Description** |
| dx_n_B02.2 | Number of ICD10 Code Zoster with other nervous system involvement |
| dx_n_B02.8 | Number of ICD10 Code Zoster with other complications |
| dx_n_B02.9 | Number of ICD10 Code Zoster without complications |
| dx_n_D48.1 | Number of ICD10 Code Neoplasm of uncertain behavior of connective and other soft tissue |
| dx_n_E11.4 | Number of ICD10 Code Type 2 diabetes mellitus with diabetic neuropathy, unspecified |
| dx_n_F32.9 | Number of ICD10 Code Major depressive disorder, single episode, unspecified |
| dx_n_F41.0 | Number of ICD10 Code Panic disorder [episodic paroxysmal anxiety] |
| dx_n_F41.1 | Number of ICD10 Code Generalized anxiety disorder |
| dx_n_F41.8 | Number of ICD10 Code Other specified anxiety disorders |
| dx_n_F41.9 | Number of ICD10 Code Anxiety disorder, unspecified |
| dx_n_F43.1 | Number of ICD10 Code Post-traumatic stress disorder (PTSD) |
| dx_n_F43.2 | Number of ICD10 Code Adjustment disorders |
| dx_n_F43.9 | Number of ICD10 Code Reaction to severe stress, unspecified |
| dx_n_G43.7 | Number of ICD10 Code Chronic migraine without aura |
| dx_n_G43.9 | Number of ICD10 Code Migraine, unspecified |
| dx_n_G44.2 | Number of ICD10 Code Tension-type headache |
| dx_n_G44.5 | Number of ICD10 Code Complicated headache syndromes |
| dx_n_G50.0 | Number of ICD10 Code Disorders of trigeminal nerve |
| dx_n_G54.0 | Number of ICD10 Code Brachial plexus disorders |
| dx_n_G56.0 | Number of ICD10 Code Carpal tunnel syndrome |
| dx_n_G56.1 | Number of ICD10 Code Other lesions of median nerve |
| dx_n_G56.2 | Number of ICD10 Code Lesion of ulnar nerve |
| dx_n_G56.3 | Number of ICD10 Code Lesion of radial nerve |
| dx_n_G56.4 | Number of ICD10 Code Causalgia of upper limb |
| dx_n_G57.0 | Number of ICD10 Code Lesion of sciatic nerve |
| dx_n_G57.1 | Number of ICD10 Code Meralgia paresthetica |
| dx_n_G57.3 | Number of ICD10 Code Lesion of lateral popliteal nerve |
| dx_n_G57.5 | Number of ICD10 Code Tarsal tunnel syndrome |
| dx_n_G57.6 | Number of ICD10 Code Lesion of plantar nerve |
| dx_n_G57.7 | Number of ICD10 Code Causalgia of lower limb |
| dx_n_G57.8 | Number of ICD10 Code Other specified mononeuropathies of lower limb |
| dx_n_G57.9 | Number of ICD10 Code Unspecified mononeuropathy of lower limb |
| dx_n_G58.9 | Number of ICD10 Code Mononeuropathy, unspecified |
| dx_n_G89.2 | Number of ICD10 Code Chronic pain, not elsewhere classified |
| dx_n_G89.3 | Number of ICD10 Code Neoplasm related pain (acute) (chronic) |
| dx_n_G89.4 | Number of ICD10 Code Chronic pain syndrome |
| dx_n_I10 | Number of ICD10 Code Essential (primary) hypertension |
| dx_n_I20.0 | Number of ICD10 Code Unstable angina |
| dx_n_I20.1 | Number of ICD10 Code Angina pectoris with documented spasm |
| dx_n_I20.8 | Number of ICD10 Code Other forms of angina pectoris |
| dx_n_I20.9 | Number of ICD10 Code Angina pectoris, unspecified |
| dx_n_I73.0 | Number of ICD10 Code Raynaud's syndrome |
| dx_n_M17.1 | Number of ICD10 Code Unilateral primary osteoarthritis of knee |
| dx_n_M25.4 | Number of ICD10 Code Effusion of joint |
| dx_n_M25.5 | Number of ICD10 Code Pain in unspecified joint |
| dx_n_M25.7 | Number of ICD10 Code Osteophyte |
| dx_n_M26.0 | Number of ICD10 Code Major anomalies of jaw size |
| dx_n_M47.2 | Number of ICD10 Code Other spondylosis with radiculopathy |
| dx_n_M47.8 | Number of ICD10 Code Other spondylosis |
| dx_n_M48.0 | Number of ICD10 Code Spinal stenosis |
| dx_n_M48.1 | Number of ICD10 Code Ankylosing hyperostosis [Forestier] |
| dx_n_M50.1 | Number of ICD10 Code Cervical disc disorder with radiculopathy |
| dx_n_M51.1 | Number of ICD10 Code Thoracic, thoracolumbar and lumbosacral intervertebral disc disorders with radiculopathy |
| dx_n_M53.8 | Number of ICD10 Code Other specified dorsopathies |
| dx_n_M54.1 | Number of ICD10 Code Radiculopathy |
| dx_n_M54.2 | Number of ICD10 Code Cervicalgia |
| dx_n_M54.5 | Number of ICD10 Code Low back pain |
| dx_n_M54.8 | Number of ICD10 Code Other dorsalgia |
| dx_n_M54.9 | Number of ICD10 Code Dorsalgia, unspecified |
| dx_n_M60.8 | Number of ICD10 Code Other myositis |
| dx_n_M62.2 | Number of ICD10 Code Nontraumatic ischemic infarction of muscle |
| dx_n_M62.4 | Number of ICD10 Code Contracture of muscle |
| dx_n_M62.8 | Number of ICD10 Code Other specified disorders of muscle |
| dx_n_M65.1 | Number of ICD10 Code Other infective (teno)synovitis |
| dx_n_M65.3 | Number of ICD10 Code Trigger finger |
| dx_n_M65.4 | Number of ICD10 Code Radial styloid tenosynovitis [de Quervain] |
| dx_n_M65.8 | Number of ICD10 Code Other synovitis and tenosynovitis |
| dx_n_M67.3 | Number of ICD10 Code Transient synovitis |
| dx_n_M70.2 | Number of ICD10 Code Olecranon bursitis |
| dx_n_M70.3 | Number of ICD10 Code Other bursitis of elbow |
| dx_n_M70.6 | Number of ICD10 Code Trochanteric bursitis |
| dx_n_M70.7 | Number of ICD10 Code Other bursitis of hip |
| dx_n_M71.3 | Number of ICD10 Code Other bursal cyst |
| dx_n_M72.8 | Number of ICD10 Code Other fibroblastic disorders |
| dx_n_M75.8 | Number of ICD10 Code Other shoulder lesions |
| dx_n_M76.0 | Number of ICD10 Code Gluteal tendinitis |
| dx_n_M76.1 | Number of ICD10 Code Psoas tendinitis |
| dx_n_M76.2 | Number of ICD10 Code Iliac crest spur |
| dx_n_M76.3 | Number of ICD10 Code Iliotibial band syndrome |
| dx_n_M76.6 | Number of ICD10 Code Achilles tendinitis |
| dx_n_M76.7 | Number of ICD10 Code Peroneal tendinitis |
| dx_n_M76.8 | Number of ICD10 Code Other specified enthesopathies of lower limb, excluding foot |
| dx_n_M77.0 | Number of ICD10 Code Medial epicondylitis |
| dx_n_M77.1 | Number of ICD10 Code Lateral epicondylitis |
| dx_n_M77.4 | Number of ICD10 Code Metatarsalgia |
| dx_n_M77.5 | Number of ICD10 Code Other enthesopathy of foot and ankle |
| dx_n_M77.9 | Number of ICD10 Code Enthesopathy, unspecified |
| dx_n_M79.1 | Number of ICD10 Code Myalgia |
| dx_n_M79.2 | Number of ICD10 Code Neuralgia and neuritis, unspecified |
| dx_n_M79.6 | Number of ICD10 Code Pain in limb, hand, foot, fingers and toes |
| dx_n_M79.7 | Number of ICD10 Code Fibromyalgia |
| dx_n_M79.8 | Number of ICD10 Code Other specified soft tissue disorders |
| dx_n_M96.1 | Number of ICD10 Code Postlaminectomy syndrome, not elsewhere classified |
| dx_n_M99.3 | Number of ICD10 Code Osseous stenosis of neural canal |
| dx_n_M99.4 | Number of ICD10 Code Connective tissue stenosis of neural canal |
| dx_n_M99.5 | Number of ICD10 Code Intervertebral disc stenosis of neural canal |
| dx_n_M99.6 | Number of ICD10 Code Osseous and subluxation stenosis of intervertebral foramina |
| dx_n_M99.7 | Number of ICD10 Code Connective tissue and disc stenosis of intervertebral foramina |
| dx_n_R10.9 | Number of ICD10 Code Unspecified abdominal pain |
| dx_n_R51.9 | Number of ICD10 Code Headache, unspecified |
| dx_n_R53.8 | Number of ICD10 Code Other malaise and fatigue |
| dx_n_S14.3 | Number of ICD10 Code Injury of brachial plexus |
| dx_n_Z51.1 | Number of ICD10 Code Encounter for antineoplastic chemotherapy and immunotherapy |
| dx_n_Z51.8 | Number of ICD10 Code Encounter for other specified aftercare |
| dx_n_Z79.1 | Number of ICD10 Code Long term (current) use of non-steroidal anti-inflammatories (NSAID) |
| dx_n_Z79.8 | Number of ICD10 Code Other long term (current) drug therapy |
| dx_n_Z92.2 | Number of ICD10 Code Personal history of drug therapy |
| dx_n_Z96.6 | Number of ICD10 Code Presence of orthopedic joint implants |
| sa_n | Number of Substance Abuse Diagnosis Codes |
| **Provider Notes Department History** |  |
| **Factors** | **Description** |
| notes_n_ANE | Number of Notes w/ Anesthesiology |
| notes_n_CIM | Number of Notes w/ Community Internal Medicine |
| notes_n_CM | Number of Notes w/ Care Management |
| notes_n_CVD | Number of Notes w/ Cardiovascular Diseases |
| notes_n_DER | Number of Notes w/ Dermatology |
| notes_n_ED | Number of Notes w/ Emergency Medicine |
| notes_n_END | Number of Notes w/ Endocrinology |
| notes_n_FAM | Number of Notes w/ Family Medicine |
| notes_n_GIH | Number of Notes w/ Gastroenterology and Hepatology |
| notes_n_HIM | Number of Notes w/ Hospital Internal Medicine |
| notes_n_NES | Number of Notes w/ Neurological Surgery |
| notes_n_NEU | Number of Notes w/ Neurology |
| notes_n_NSG | Number of Notes w/ Nursing Services |
| notes_n_ONC | Number of Notes w/ Oncology |
| notes_n_ORS | Number of Notes w/ Orthopedic Surgery |
| notes_n_PHR | Number of Notes w/ Pharmacy |
| notes_n_PMR | Number of Notes w/ Physical Medicine and Rehabilitation |
| notes_n_PSYPSI | Number of Notes w/ Psychiatry and Psychology |
| notes_n_PTO | Number of Notes w/ Physical and Occupational Therapy |
| notes_n_RAD | Number of Notes w/ Radiology |
| notes_n_RHU | Number of Notes w/ Rheumatology |
| notes_n_SPN | Number of Notes w/ Spine |
| notes_n_URO | Number of Notes w/ Urology |
| **NLP concepts** |  |
| **Concept Names** | **Corresponding Procedure** |
| AMPUTEE | PNS |
| AXILLARY NERVE | PNS |
| CRYOABLATION | PNS |
| FEMORAL NEUROPATHY | PNS |
| GENITOFEMORAL NERVE | PNS |
| ILIOINGUINAL NEURALGIA | PNS |
| LATERAL FEMORAL CUTANEOUS NEUROPATHY | PNS |
| MEDIAN NERVE | PNS |
| NEUROMA PAIN | PNS |
| POSTHERPETICN EURALGIA | PNS |
| PULSED RADIOFREQUENCY | PNS |
| RADIALNERVE | PNS |
| SAPHENOUS NERVE | PNS |
| SCIATIC NEUROPATHY | PNS |
| SURAL NERVE | PNS |
| TIBIAL NEUROPATHY | PNS |
| ULNAR NEUROPATHY | PNS |
| MONONEUROPATHY | PNS |
| HERNIORRHAPHY | PNS |
| PNS | PNS |
| NERVE INJURY | PNS / SCS |
| ALLODYNIA | SCS |
| ANGINA | SCS |
| COMPLEX REGIONAL PAIN SYNDROME | SCS |
| DIABETIC NEUROPATHY | SCS |
| DISCECTOMY | SCS |
| FAILED BACK SURGERY SYNDROME | SCS |
| FUSION RELATED | SCS |
| NEUROMODULATION | SCS |
| NEUROPATHICPAIN | SCS |
| NEUROPATHICPAINFOOT | SCS |
| NEUROPATHICPAINHAND | SCS |
| PERIPHERAL NEUROPATHY | SCS |
| PERIPHERAL VASCULAR DISEASE | SCS |
| RADICULOPATHY | SCS |
| ARACHNOIDITIS | SCS |
| SCS | SCS |

Appendix Table2 Summary of Strengths and Weaknesses of Models

| **Model** | **Strengths** | **Weaknesses** |
| --- | --- | --- |
| **Easy Ensemble [22]** | - Combines multiple balanced subsets using Adaboosting, improving minority class detection.  - can handle severe imbalance effectively. | - Computationally expensive due to multiple model training. |
| **Balanced Bagging [23]** | - Resamples minority and majority classes within each base learner to maintain balance and improve minority recognition.  - Applicable to both binary and multiclass classification | - May still underperform if base learners are weak. |
| **RUSBoost [24]** | - Effective for high imbalance ratios by randomly removing majority samples to balance the data in each boosting iteration .  - Reduces computational cost compared to Easy Ensemble. | - Random under-sampling may discard informative majority samples |
| **Balanced Random Forest [25]** | - Forces each tree to see a balanced set of samples, improving detection of minority cases. | - Can increase variance and reduce overall accuracy on the majority class |
| **Random Forest [26]** | - Handles complex feature interactions and nonlinear relationships effectively. | - Still somewhat biased toward the majority class in extremely imbalanced datasets even after balanced class weight. |
| **Logistic Regression(L1/L2)** | - Easy to understand and explain.  - L1 regularization can perform feature selection, improving model sparsity. | - Finds simple patterns, not good for complex non-linear relationships. |
| **One-Class SVM [27]** | - Good for finding rare or unusual cases | - Computationally intensive  - Can increase FPR. |
| **SVC [28]** | - Good for finding complex decision boundaries. | - Computationally intensive |
