# Supplementary material for: Multistage machine learning model for automated referral triage in pain medicine
Source: Future Healthc J. 2026 Jan 6;13(1):100500. doi: 10.1016/j.fhj.2026.100500 (PMC12860341; doi:10.1016/j.fhj.2026.100500)
Supplement: Supplementary file 2 [file mmc2.pdf]

Supplementary Material

Additional Testing

The additional 6-month (from January 2024 to June 2024) testing results provided a view of model performance on the temporal data; see Table S1. Overall, most procedures showed improved accuracy, driven primary by gains in TNR. This pattern differed from the 10-fold cross-validation results, where gains in TPR were more pronounced. A key reason was that Easy Ensemble with AdaBoost used a loss function that weighed all errors equally while learning. Thus, it was easier for the model to prioritize reducing errors for negative cases, the majority, since there were not enough positive cases to improve further in sensitivity especially on PNS, MILD, and BVRF. It is also expected that temporal testing performance may not fully match cross-validation results. The reason could be during this six-month referral patterns may have changed; see Table S2. While acknowledging these limitations, we believe the model's triage accuracy could provide incremental value to current clinical practice. To be noted, increasing true negative (reducing false positive) is as important from resource utilization standpoint. Further investigations into testing the proposed model are needed.

Top 10 Features of Final Stage Models

Table S2 summarizes the top 10 most important features of the final-stage model for each procedure. The top features across all procedures showed both clinical relevance and predictive value. The previous stage's predicted probability consistently ranked the highest, confirming the benefit of the multistage learning approach. For PNS that required three stages, both previous stages probabilities were listed as top features. Some of important features in our findings align with what was reported in literature, which emphasizes that age, diagnostic patterns, and multidisciplinary care utilization are important predictors of neuromodulation. While some important features align with established clinical reasoning, other features such as the number of other medication orders, additional diagnoses, or procedures, might reflect patient complexity (e.g., comorbid symptoms, failed conservative therapy) and the needs for patient visits. This variability reflected a key advantage of machine learning: its ability to detect hidden or nonlinear relationships within complex clinical data that may not be immediately apparent through conventional clinical observation. Additionally, Table S2 indicated the changes in feature importance between the training and temporal testing periods, providing insight into shifts in model behavior over time. While several key predictors showed consistent average values across both datasets, some variables demonstrated notable changes in the testing cohort. For SCS, the positive cases for number of other Hierarchical Condition Category History (HCC), Radiculopathy, Dorsalgia, Spinal stenosis increased significantly in the 6-month testing dataset. For PNS, the positive cases also increased substantially in testing data for number of medication orders, notes and appointments with Orthopedic Surgery, appointments with Radiology, and number of other HCC. Similar behaviors were also exhibited in testing data for other procedures. These shifts likely reflected changes in patient patterns and clinical documentation behaviors during the subsequent six-month period. Such differences contributed to the observed variation in model performance and highlighted the importance of ongoing monitoring after deploying such models.

Table S1. 6-Month Testing Result Summary

		First Stage				*Final Stage*			
**Procedure**	**Positive Cases**	**TPR**	**TNR**	**Accuracy**	**AUC**	**TPR**	**TNR**	**Accuracy**	**AUC**
**SCS**	21	76.7%	74.9%	75.0%	0.83	80.0%	72.3%	72.5%	0.82
**ITP**	4	100.0%	88.2%	88.3%	0.98	100.0%	96.1%	96.2%	0.99
**PNS**	12	83.3%	80.3%	80.3%	0.91	58.3%	88.5%	88.0%	0.67
**BVRF**	5	80.0%	72.1%	72.2%	0.78	40.0%	95.1%	94.8%	0.71
**MILD**	6	83.3%	81.7%	81.7%	0.91	33.3%	95.9%	95.4%	0.66

Table S2. Top 10 Most Important Features of The Final-Stage models for Each Procedure

Procedure	Top 10 important features	Training Data (mean) 0	Training Data (mean) 1	Testing Data (mean) 0	Testing Data (mean) 1
SCS	1st stage predicted probability	0.47	0.56	0.47	0.54
	Age	59.91	61.29	60.51	61.72
	Number of Medication Order for Other	25.50	25.89	23.72	22.19
	Number of Appointments w/ Radiology	8.63	8.92	8.06	6.81
	Number of Notes w/ cardiovascular diseases	2.44	3.21	2.08	3.62
	Number of Other HCC history	81.57	70.92	175.55	116.86
	Number of ICD10 Code Radiculopathy	1.19	2.58	3.85	10.81
	Number of ICD10 Code Dorsalgia, unspecified	0.34	0.59	0.58	2.76
	Number of ICD10 Code Spinal stenosis	0.90	1.07	2.39	6.81
	Number of Medication Order for Opioid	3.63	4.35	2.86	2.90
PNS	2nd stage predicted probability	0.41	0.62	0.40	0.55
	Age	59.97	59.27	60.48	64.43
	1st stage predicted probability	0.48	0.57	0.47	0.55
	Number of Medication Order for Other	25.57	20.85	23.25	50.92
	Number of Notes w/ Orthopedic Surgery	6.68	11.74	3.92	28.00
	Number of Appointments w/ Other	26.24	20.70	22.33	41.08
	Number of Other Procedures	0.40	1.30	0.21	0.58
	Number of Appointments w/ Radiology	8.66	7.11	7.76	24.67
	Number of Other HCC history	81.46	60.52	169.33	472.33
	other_appts_n_Orthopedic.Surgery	1.82	3.65	1.24	9.33
BVRF	1st stage predicted probability	0.45	0.57	0.46	0.55
	Age	59.99	54.40	60.61	50.45
	Number of Medication Order for Other	25.55	16.63	23.73	14.80
	Number of Appointments w/ Radiology	8.66	3.38	8.05	3.40
	Number of Notes w/ cardiovascular diseases	2.48	0.06	2.14	0.00
	Number of Appointments w/ Other	26.20	19.75	22.66	15.40
	Number of ICD10 Code Essential (primary) hypertension	1.66	0.00	3.25	2.20
	Number of ICD10 Code Dorsalgia, unspecified	0.35	0.13	0.64	0.00
	Number of Notes w/ Nursing Services	5.41	1.25	5.05	2.40
	Gender n (%)	1978 (55.9%)	6 (37.5%)	466 (57.5%)	2 (45.5%)
ITP	1st stage predicted probability	0.43	0.58	0.43	0.60
	Number of Appointments w/ Neurology	1.05	2.08	1.02	0.50
	Number of Medication Order for Other	25.36	40.83	23.53	52.75
	Number of Notes w/ Neurology	2.28	5.97	1.89	5.75
	Age	60.02	54.14	60.56	57.04
	Number of Notes w/ Anesthesiology	2.73	3.28	3.02	9.25
	Number of Notes w/ Family Medicine	10.88	22.97	7.63	17.25
	Number of Notes w/ Physical Medicine and Rehabilitation	1.84	16.53	1.62	13.25
	Number of Appointments w/ Other	26.15	28.19	22.51	43.00
	Number of Appointments w/ Other Surgery	0.80	0.67	0.53	1.00
MILD	1st stage predicted probability	0.36	0.62	0.38	0.61
	Number of Other Procedures	0.41	1.11	0.21	0.17
	HCC history of Chronic Kidney Disease Mild / Unspecified / Stages 1 or 2 / Unspecified	1.30	1.44	2.17	8.17
	Number of Notes w/ Anesthesiology	2.74	1.33	3.06	2.83
	Number of Appointments w/ Orthopedic Surgery	1.84	2.33	1.37	0.17
	Number of ICD10 Code Pain in limb, hand, foot, fingers and toes	1.65	0.78	3.24	6.83
	Age	59.91	80.23	60.39	80.35
	Number of Medication Order for Duloxetine	0.43	1.56	0.40	0.17
	Number of Notes w/ Spine	0.90	6.44	0.68	1.17
	Number of Medication Order for Blood Thinner	0.96	1.11	0.76	0.33
